# The Value of Darkness: A Moral Framework for Urban Nighttime Lighting

**DOI:** 10.1007/s11948-017-9924-0

**Published:** 2017-06-08

**Authors:** Taylor Stone

**Affiliations:** Ethics and Philosophy of Technology Section, Delft University of Technology, Jaffalaan 5, 2628 BX Delft, The Netherlands

**Keywords:** Darkness, Light pollution, Artificial light at night, Nighttime illumination, Design for values, Environmental ethics

## Abstract

The adverse effects of artificial nighttime lighting, known as *light pollution*, are emerging as an important environmental issue. To address these effects, current scientific research focuses mainly on identifying what is bad or undesirable about certain types and uses of lighting at night. This paper adopts a value-sensitive approach, focusing instead on what is good about darkness at night. In doing so, it offers a first comprehensive analysis of the environmental value of darkness at night from within applied ethics. A *design for values* orientation is utilized to conceptualize, define, and categorize the ways in which value is derived from darkness. Nine values are identified and categorized via their type of good, temporal outlook, and spatial characteristics. Furthermore, these nine values are translated into prima facie moral obligations that should be incorporated into future design choices, policy-making, and innovations to nighttime lighting. Thus, the value of darkness is analyzed with the practical goal of informing future decision-making about urban nighttime lighting.

## Introduction: Darkness at Night as a Moral Issue

Cities at night evoke a variety of images, ranging from a perilous time filled with nefarious characters to the vibrancy and excitement of nighttime entertainment. Here a novel issue of increasing importance to cities at night will be analyzed: the evolving morality of darkness. In recent years the negative impacts of artificial nighttime lighting have come under scrutiny, and the concept of *light pollution* has become the dominant driver of this discourse. Resultant policy-oriented research largely focuses on delineating the bad or detrimental aspects of artificial light at night, while saying very little about the goodness of a lack of light. Current literature is missing a comprehensive account of the positive value of darkness at night, and an understanding of how this can be incorporated into the design of nighttime lighting. Often, darkness is assumed to be antagonistic to the benefits of artificial lighting, or a neutral backdrop for the possibilities created by nighttime illumination. Alternatively, darkness is seen as similar to other natural features: as something valuable “out there,” to be experienced in parks and wilderness reserves. However, current debates over light pollution provide an opportunity to re-examine darkness at night and ask why (and how) we should protect or re-introduce darkness into our urban nightscapes. Towards this goal, a first comprehensive analysis of the value of darkness, as related to decision-making about urban nighttime lighting, is provided. This paper will conceptualize, define, and categorize the value of darkness at night from an environmental perspective, providing a detailed account of the goodness being degraded or hindered by light pollution. In doing so, a framework is introduced that goes beyond reducing the negative effects of lighting, aspiring to promote and preserve what is valuable about darkness. Thus, it puts designers and policy-makers in a better position to make informed, value-sensitive decisions about urban nighttime lighting. As such, this framework is best understood as being constructively critical of, and ultimately complimentary to, the existing framing offered by the concept of light pollution.

Concerns about light pollution are often articulated as a *loss of the night* (e.g. Bogard 2013), but what exactly are we losing? In a world of abundant and increasing artificial lighting, a better understanding of why we should protect or preserve darkness is urgently needed. A philosophical, and especially moral, investigation into the disappearance of darkness should be seen as a pragmatic endeavor. Falchi et al. (2016) recently published an updated “world atlas of artificial night sky brightness,” concluding that 83% of the world’s population, and over 99% of people living in Europe and the United States, live in places with a sky considered to be light-polluted (a minimum of about an 8% increase above natural nighttime conditions). In densely populated urban regions, artificial brightness is often several magnitudes greater. And, it is estimated that artificial nighttime brightness is increasing annually by around 3–6% worldwide (Hölker et al. 2010). Faced with such a reality, there is a need to move beyond a metaphysical reflection on the nature and meaning of darkness and look critically at our current context. This means assessing if (or why) the disappearance of darkness is bad, and why its preservation is good. And, it means considering how this should factor into the articulation of morally acceptable uses of artificial lighting at night.

This paper is focused on two interrelated goals: articulating a pluralistic, value-sensitive understanding of darkness relevant to contemporary discourse, and a detailed analysis of the ways in which darkness can be valued—and ultimately operationalized—from an environmental perspective. “The value of investigating the value of darkness” section discusses the context of this analysis in more detail, explaining the shortcomings of the concept of light pollution and the usefulness of a *design for values* approach. The “Articulating the value of darkness” section then articulates a pragmatic view of the value of darkness that is sensitive to the influence of nighttime lighting technologies. In the “Categorizing the value of darkness” section nine valuations of darkness are identified and defined, which are then compared and categorized based on three criteria: type of good, temporal characteristics, and spatial characteristics. The “Applying the value of darkness: from evaluation towards prescription” section considers how this framework can be utilized as a tool for decision-making. The nine valuations of darkness are translated into prima facie moral obligations, and the prescriptive potential of this framework is shown via a brief critique of the increasingly popular adoption of LED streetlights.

## The Value of Investigating the Value of Darkness

The following section provides further context for this analysis. First, a brief discussion of the concept of light pollution (and its shortcomings) is presented. Next, the theoretical orientation of *design for values* is articulated in light of the present endeavor.

### Beyond the Concept of Light Pollution

Properly assessing contemporary evaluations of darkness requires an understanding of the concept that currently shapes discourse: light pollution. More generally, it must be appreciated that any discussion of darkness at night is also a discussion of lighting. The modern history of the night is largely a history of developments in artificial nighttime lighting and the technological, social, economic, and spatial changes it brought about (e.g., Melbin 1987; Nye 1990; Schivelbusch 1988; Schlör 1998). It is no surprise, then, that when concerns emerged about the effects of artificial lighting at night, they followed this same narrative. The concept of *light pollution* emerged in the 1970s as term to encapsulate and categorize the adverse impacts of nighttime illumination (Sperling 1991), and has gained significant academic and public attention in recent years. The International Dark-Sky Association, arguably the leading authority on light pollution, defines light pollution simply as “any adverse effect of artificial light” (IDA 2014). A more nuanced articulation of the concept states, “the unintended consequences of poorly designed and injudiciously used artificial lighting are known as light pollution” (Gallaway 2010, 72). There are compelling reasons for the increasing attention on the negative impacts of artificial nighttime lighting: it wastes billions of dollars and massive amounts of energy, it is damaging habitats and biodiversity in ways we are only beginning to understand, it likely has a negative effect on human health and well-being, and it cuts off experiences of a natural night sky.^1^ Thus, it follows that existing policy-oriented work is focused on mitigating the causes and effects of light pollution, and defining acceptable uses of artificial lighting at night (e.g., Falchi et al. 2011; Hölker et al. 2010; Kyba et al. 2014; Meier et al. 2014; Mizon 2012).

With the growing recognition that light pollution is a pressing urban and environmental issue of the twenty first century, we will increasingly be faced with complex moral and political debates about responsible uses of, and technological innovations to, artificial lighting at night. Elsewhere, I discuss the effectiveness of the concept of light pollution in framing the environmental problems—and potential solutions—of artificial nighttime illumination (Stone forthcoming). A practical outlook was adopted, with the idea that an “upstream” ethical analysis can contribute to later decision-making and policy choices (Elliott 2009). In taking this approach, two important criticisms were highlighted. First, light pollution—as a prescriptive moral concept—is limited, as it only delineates bad types (and effects) of lighting while saying very little about what good lighting is (save for it being absent of adverse effects). Second, the threshold or boundary conditions for lighting deemed to be “polluting” is often ambiguous, and the evaluative foundations and mechanisms for such a categorization require further clarification.

This paper works to address the shortcomings of the concept of light pollution by providing a conceptual analysis and categorization of what is good about a lack of light at night. When considered from a values-level perspective, focusing solely on negative consequences gives an incomplete picture. Historical studies illustrate that the development of nighttime illumination has shaped, and been shaped by, various social values, such as safety, prosperity, and progress (e.g., Ekirch 2005; Nye 1990; Schivelbusch 1988). Arguments against light pollution likewise rest on an appeal to value claims, albeit from an environmental perspective; technical studies are intertwined with evaluative judgments about where and when artificial nighttime lighting should or should not be. The aim here is to tease out and explicate what those value claims are. In doing so, this framework effectively flips the discussion on light pollution away from what is wrong about artificial light at night, and towards what is good about not having so much light. This is accomplished by moving away from an analysis of why we should have less artificial light, and instead asking why we should have more darkness. A way to realize this task is to understand what is valuable about darkness at night. For this, a theoretical orientation focused on values in engineering and design is useful.

### Designing for (New) Values

A *design for values* approach is utilized, which takes the incorporation of moral values as a primary goal for design. It contends with the traditional view of design as a purely technical and value-neutral process, instead asserting that moral and societal values are inextricably linked to the design process and outcome (van den Hoven et al. 2015). The potential of a value-sensitive approach is that, by articulating what values we seek to achieve and incorporating them into design requirements and processes, we can help to create nighttime lighting infrastructure that is socially and environmentally acceptable. Such an approach resonates with the view that sustainable or ecological design has a moral requirement to go beyond simply mitigating the bad stuff—it should also strive to do more good (e.g., Buchanan 2005; McDonough and Braungart 2002; Wines 2005).

Such an analysis requires that we look past our artificial lights and explore the goodness, or valuableness, of more darkness. This requires an exploration of new moral terrain. However, this does *not* imply that darkness has yet to be given serious attention, for—as it will become clear—this analysis builds on an existing foundation of empirical research and a small but important body of environmental literature on the subject. Rather, this is meant to highlight that ideas about darkness, similar to other environmental values, are still in an *originary* stage—the time during which characteristics are “only beginning to be constituted and consolidated” (Weston 1996, 147). This requires a different sort of ethical analysis, focused more on the elucidation of value claims rather than the application of an established normative position. This is challenging, because “Operating within a culture in which certain basic values are acknowledged, at least verbally, by nearly everyone, there is little practical need to raise the question of the ultimate origins or warrants of values” (Weston 1996, 144). The origin of the concept of light pollution is a worthwhile study in itself, and one that I discuss elsewhere (Stone forthcoming). Here, the task is to identify, dissect, and systematize the value of darkness. Thus, defining and categorizing the value of darkness is, to use Weston’s (1996) phrasing, exploring an emerging issue before it hardens into an “analytic category.”

A better understanding of darkness now will provide an important step towards establishing the conditions for morally desirable nighttime lighting infrastructure; it will help to establish how we should light our twenty first century nightscapes. Epting (2016), in assessing the moral dimensions of infrastructure, proposes that their complexity necessitates a “supplemental measure” in addition to traditional moral theory. A supplementary consideration—here the value of darkness—allows for a better articulation of morally desirable outcomes for large-scale, multi-faceted urban infrastructures. Like Epting (2016), here no definitive position it taken regarding how to achieve these outcomes. Rather, an evaluative moral tool is presented that can be utilized and applied via different moral theories. Promoting and preserving darkness at night can be coupled with the mitigation of light pollution to help assess current lighting strategies, as well as foresee issues with new policies and future technological innovations. With this elucidation of darkness, we will be in a better position to analyze, judge, and ultimately make value-sensitive decisions.

## Articulating the Value of Darkness

This section will further elaborate on the meaning of darkness applied in this paper. Before going further, though, a quick note on terminology is required. *Darkness* and *night* are closely linked, but not synonymous. *Night* is too broad and indefinite to be seen as valuable in any meaningful sense. *Darkness*, alternatively, is a central feature of the night, and one that we—by way of artificial lighting technologies—have the capacity to influence. Literature on light pollution refers to a similar claim while using different terminology: the night sky, natural nights, natural nighttime conditions, etc. I see *darkness* as most appropriate given its achievability via technical means, as well as the related quantitative and qualitative control that lighting technologies have on when, where, and how much darkness to (re)introduce into our urban nights.

### Foundations of Valuing Darkness

Historically, darkness has been seen as full of evil spirits, chaotic and dangerous, a space and time for immoral behavior, and primitive in the face of new technologies—what Edensor (2015) summarizes as our “nyctophobic” past. For centuries, darkness was largely seen as dangerous and necessitating control and domination (Schlör 1998), and later as antithetical to progress (Nye 1990). In sum, we have inherited a narrative that champions the expansion of artificial nighttime illumination:

Our image of night in the big cities is oddly enough determined by what the historians of lighting say about *light*. Only with artificial light, they tell us, do the contours of the nocturnal city emerge: the city is characterized by light. From this perspective the history of the city is a history of progressive illumination. Night is inevitably expelled into the realm of prehistory and mythology. None of the many histories of lighting, which in their different ways all describe the triumph of light, is able to dispense with a preliminary description of the impenetrable terrain of the nocturnal as an alien region of fear that is conquered and finally subjugated. (Schlör 1998, 57)

The modern history of nighttime illumination begins with the organization and formalization of public lighting projects in the seventeenth century. In reality, though, nights remained relatively dark for some time. Outdoor lighting was often only used for a few hours a night, and only on major thoroughfares (Schivelbusch 1988). However, with the invention and proliferation of gaslight and later electric light throughout the nineteenth and early twentieth centuries, nighttime illumination reached an unprecedented scale. With electric lighting, the popular ideals of *turning night into day* and *lengthening the day* became achievable in ways never before possible. As a result perceptions of darkness at night underwent a profound transformation during this time, as artificial illumination became the expectation of urban nights (Isenstadt 2014; Nye 1990). It is in this context—a world of abundant and readily available artificial light—that darkness gradually shifted from a “forbidding everyday occurance” and an “emblem of backwardness” to a valorized and “sought-after luxury” of our electrified nights (Hasenöhrl 2014, 119).

Contemporary responses to the ubiquity of artificial illumination vary, although typically focus on concerns over a *loss of the night*. Some advocate for an increase in darkness for its instrumental value (e.g. Gallaway 2014), while others highlight the underappreciated cultural and environmental losses that will result from its disappearance (e.g. Bogard 2013). Others go further still, proposing that access to dark or natural nights should be an inalienable right of all people (Starlight Initiative 2007). Taken together, there is an emerging perspective increasingly shaping, and shaped by, our modern, highly technified and illuminated nightscapes.

### Axiological and Ontological Dimensions of Darkness

It is important to briefly clarify what is meant here by “value,” as *design for values* has been criticized for lacking a precise definition (Manders-Huits 2011). At a general level, a value is something sought to be preserved, protected, pursued, or promoted (Alfano 2016). Values are not concrete objects, but rather abstract ways of understanding our relation to the world. Put more provocatively, “There are no such *things* as values. There are rather the various ways in which individuals, processes and places matter, our various modes of relating to them, and the various considerations that enter into our deliberations about action” (O’Neill et al. 2008, 1). Here the value of darkness is similarly approached as relational—deeply entwined with perceptions, interactions, and technologies. Furthermore, the value of darkness is considered to be highly contextual, both geographically and temporally. This analysis is situated in the context of our early twenty first century nightscapes in developed regions, where the ubiquity of artificial lighting (and the related disappearance of darkness) has become an inescapable reality. This requires questioning what contemporary values of environmental importance are at stake in discussions about light pollution and darkness. Thus, the focus here is on articulating what is *valuable* about darkness in our present context, and not arriving at a fundamental understanding of darkness *as a value*.

In analyzing the valuable-ness of darkness, this paper will not take a reductive approach that relates arguments to an overarching or meta-level principle for adjudicating and evaluating its moral worth, but rather give close attention to real-world complexities (Norton 1996). In this sense, it will be an examination of how different values manifest via darkness. Often, environmental debates are as much about intra-value conflicts as inter-value conflicts—an issue that has been identified as being particularly important for a *design for values* approach (Dignum et al. 2016). Analyzing the different facets of darkness will help to clarify its potential manifestation in norms or design requirements, and the conflicts and opportunities that could arise therein.

Such an analysis must be sensitive to the interrelated axiological and ontological dimensions of darkness. On one hand, it is the unifying, fundamental characteristic of our nightscapes, the base from which a multiplicity of experiences and meanings emerge. Taken in this way, darkness is not a concrete *thing* but rather an evaluative consideration that directs understandings of, and relationship with, the world at night. Yet at the same time it is a real, tangible thing accessible to direct experience. Thus, it can also be seen as a surface-level, achievable goal, as it is the condition that must be obtained or preserved to bring about desired ends. To say there is value in the ability to see the Milky Way, or alternatively an efficient and responsible use of nighttime lighting for the purpose of energy reduction, or a decrease in the deaths of migratory birds from light pollution, or a mitigation of the unwanted health effects caused by obtrusive nighttime lights, implies that darkness has value (and those things that needlessly eliminate darkness should be seen as bad in some way, or to some degree). Considered in this way, focusing on the value of darkness gives form and direction to the evaluation of our nightscapes, and provides a novel vantage point for assessing the morality of nighttime lighting. To say “the value of darkness,” then, is a convenient shorthand for a complex field of moral valuation. And it is the elucidation and categorization of this field that is the focus of the remainder of this paper.

## Categorizing the Value of Darkness

The theoretical account above positions darkness as relational, contextual, and multi-faceted. What follows is an expansion and application of this perspective via a categorization of darkness in relation to environmental concerns. As such, it reflects the existing landscape of empirical research into the effects of light pollution. It gives form and clarity to the *goodness* of darkness, as a pre-cursor to establishing how one could strive for its protection, promotion, or preservation.

### Defining the Ways Darkness is Valued

The first step is to clarify how darkness is, or could be, conceived as an environmental good. The five commonly agreed upon effects of light pollution are taken as a starting point: *energy*, *ecology*, *health*, *safety*, and the *night sky* (IDA 2014; Morgan-Taylor 2014). A review of recent literature and empirical research has expanded these categories into nine valuations of darkness (Table 1). However, it must be noted that safety will not be discussed further in this section. There are two reasons for the choice to exclude safety from this framework. First, research and discussions about safety and security at night rarely articulate darkness as *valuable*—instead, most research seeks to show that it is value-neutral by questioning the assumed relationship between more light and more safety (e.g., Bogard 2013; Gaston et al. 2015; Henderson 2010). Second, safety at night does not lead to any environmentally-relevant value of darkness. Thus, the question of where and when (and how much) lighting is useful for improved or optimal safety is a topic in itself—one that should be put in dialogue with this framework in the future, but outside of the scope of this paper. As such, questions of safety will be put aside until the conclusion.

**Table 1:** Re-framing the effects of light pollution as values of darkness

Effect of light pollution	Associated valuations of darkness
Energy	(a) Efficiency
	(b) Sustainability
Ecology	(c) Ecology
Health	(d) Healthiness
	(e) Happiness
Night sky	(f) Connection to nature
	(g) Stellar visibility
	(h) Heritage and tradition
	(i) Wonder and beauty

The four relevant effects of light pollution have been re-conceptualized as nine ways by which, or through which, environmental value is derived from darkness. Recent works giving serious consideration to questions of value and the night sky have served as useful foundations for this list.^2^ These nine valuations offer a comprehensive starting point that takes into account both the empirical work underway by biologists, economists, and astronomers, and also the qualitative arguments made by those same researchers, as well as scholars from the humanities and social sciences. They are also meant to better capture the moral arguments made against light pollution. For example, arguments against the adverse effects of artificial nighttime light to ecosystems and wildlife—while a diverse and complex field of research—appear to follow a coherent moral argument focused on conservation efforts. Comparatively, arguments for the protection of dark or natural night skies seem to rely on a more varied (if interrelated) set of moral concerns. As such, an expansion of that category was deemed appropriate. In what follows, each of the nine identified valuations are briefly defined.

- Efficiency: Outdoor nighttime lighting is a large consumer of energy. Globally, it represents 8% of total electricity consumption for lighting, estimated at 218 TWh (De Almeida et al. 2014). Furthermore, it is estimated that upwards of 30% of outdoor light is wasted in the United States, with a cost of almost $7 billion dollars annually (Gallaway et al. 2010). Likewise, an estimate of wasted light in the European Union predicts the annual costs to be around €5 billion (Morgan-Taylor 2014). Unneeded or wasteful nighttime lighting can be a particularly visible form of excessive consumption, and a reintroduction or protection of darkness becomes symbolic of the efficient use of lighting resources, only using lighting where and when it is needed. Darkness, when understood as a manifestation of efficiency, can conceivably have immense economic value, particularly in urbanized regions.
- Sustainability: While “sustainability” can be interpreted in many ways, here it is meant to invoke the inter-generational concerns of sustainable development, and in particular the reduction of energy usage as a means to combat and/or mitigate climate change. Considered in this way, the wastefulness of outdoor lighting can be associated with energy usage and greenhouse gas emissions. The use of outdoor artificial lighting is a significant contributor to greenhouse gas emissions, to the degree that cutting all wasted light in the United States could have the equivalent effect on CO_2_ emissions as removing 9.5 million cars from the road (Gallaway et al. 2010). From such a perspective, advocating for darker nights becomes a way to promote responsible energy usage and mitigate CO_2_ emissions.
- Ecology: Research indicates that nighttime lighting has profound effects on wildlife, notably migratory birds, sea turtles, and bats (Pottharst and Konecke 2013; Rich and Longcore 2005). While a diverse and complex set of issues and research is represented by this valuation, it rests on a coherent moral claim associated with the notion of “ecological light pollution” (Longcore and Rich 2004). Species and ecosystems have evolved within natural diurnal cycles that are being drastically altered by artificial light at night, especially in urbanized areas. It can be argued that naturally occurring levels of daylight and darkness at night are not only essential to the protection of species and habitats, but also inherent to their functioning and thriving. As such, the protection or re-introduction of darker nights is closely aligned with species and biodiversity protection, as well as nighttime habitat conservation efforts.
- Healthiness: Humans are also affected by excess artificial nighttime illumination. Our bodies have evolved within natural cycles of light and dark, and the relatively rapid change may have negative effects. The disruption of our circadian rhythm has been linked to reduced visibility at night, obesity, insomnia, and certain types of cancer (Chepesiuk 2009; Cho et al. 2015; Falchi et al. 2011). While understandings of these effects is still somewhat preliminary, the World Health Organization recently upgraded the exposure to certain types of light at night to the category of “likely carcinogen” (Morgan-Taylor 2014). Thus, allowing access to, and the experience of, darker nights can be seen as valuable for personal health. In this capacity, it would appear that darkness is less analogous to a broader societal value, but rather a physical characteristic that one should seek to achieve and foster towards the goal of a healthy lifestyle.
- Happiness: There can be a further distinction made between physical well-being and psychological or emotional well-being. With respect to the latter, Gallaway (2014) proposes a link between happiness and access to a natural night sky, outlining the many beneficial traits of dark nights. In this analysis, Gallaway draws on recent economic literature, as well as research from environmental psychology asserting the restorative and beneficial effects of contact with natural settings (e.g., Berman et al. 2008; Mayer et al. 2009). Gallaway (2014) posits that the night sky can contribute to happiness via factors such as: the focus on experiences rather than consumables; increasing small, regular experiences of pleasure over infrequent, intense ones; the pleasure derived from experiences of (natural) beauty; and, the relaxing and restorative powers of interactions with natural nights skies. Thus, darkness can be seen as something with the potential to facilitate and promote psychological well-being, broadly conceived.
- Connection to nature: Fostering a connection to nature, and the ability to experience natural settings, is a central concern within environmental philosophy. It is also an identified goal for some conceptions green design (e.g. Buchanan 2005; Wines 2005). *Let There Be Night* (Bogard 2008) offers a collection of reflections on the powerful experience of natural nighttime conditions, an in particular the night sky, made accessible via darkness. In this respect, darkness can be understood as analogous to, or symbolic of, natural nighttime conditions. Concerns over the *loss of the night* are not about a literal loss—the night is not going anywhere. Rather, a lack of darkness signifies the vast anthropogenic changes that have occurred to nightscapes during the last century. Artificial lighting is altering nighttime conditions, to the extant that the ability to experience natural nighttime conditions is becoming increasingly rare. Thus, promoting dark nights can be understood as a way to preserve a connection to the more-than-human world.
- Stellar visibility: A closely related concern to the notion of a “connection to nature” is the decreased visibility of starlight. In fact, this issue was an early reason for criticisms of nighttime illumination (Hasenöhrl 2014), as well as the eventual emergence of the term light pollution (Sperling 1991). In most urbanized areas you are lucky to see a few dozen stars, compared to a few thousand on a clear, dark night. This concern is often practical, as astronomical observatories have been relegated to remotely inhabited areas. However it has also emerged as an aesthetic, spiritual, and moral concern. Starlight is a central feature of natural nighttime conditions, and often invoked as the primary aspect of the night we risk losing. Darkness at night, and especially dark skies, is a precondition for access to the firmament.
- Heritage and tradition: Historically, the night sky has played a central role in various cultures and traditions across the world, having a prominent role within mythology, religion, art and literature, navigation, conceptions of time, and scientific discovery (Gallaway 2014). The *loss of the night* also implies the loss of this heritage, and darkness at night is the precondition through which continued access is possible. The preservation of the night sky, made possible by dark nights, is therefore also a preservation of this heritage for present and future generations.
- Wonder and beauty: Darkness at night, and in particular the features of the night made accessible by dark skies, is an awe-inspiring experience. To look out at the night sky is a truly sublime experience in which you instantly travel across unimaginable distances and sense a universe larger than our own world. The wonder and beauty of the night sky is often argued to be of immense value, and a value that cannot be properly captured in quantitative terms (e.g., Bogard 2013; Gallaway 2014; Henderson 2010). Thus, it is important to recognize the aesthetic appeal of the night sky—inextricably connected to the preservation and promotion of darkness.

This list offers a comprehensive overview of the ways in which darkness is valuable and likewise conceived as an environmental good. The following three sections build on these definitions by comparing their characteristics in three ways: by type of good, temporal outlook, and spatial characteristics. It should be noted that a definitive claim about the completeness of this list is not being made. It is certainly possible that an environmentally-relevant reason for valuing darkness is missing, or that an argument can be made for further separating (or combining) one or more of the above values. That said, it is postulated that any such amendment will still fit into the framework developed here, thus ultimately serving to strengthen its usefulness.

### Categorizing by Type of Good

As a first step in assessing the relationship between these nine values of darkness, a distinction can be made between those values for which darkness is *inherent*—meaning that darkness is an intrinsic quality of the desired end—and those values for which darkness is merely *instrumental* to their achievement. The main question under consideration is thus: *is darkness a means to some other end, or a component of the end itself*? The distinction comes with important implications. To achieve certain values in the context of designing nightscapes, such as a stellar visibility, darkness is inextricably linked to the desired end. It is darkness *itself* that we seek to preserve or promote, because of the good it is expected to bring about. To see darkness as intrinsically valuable attaches an increased, and arguably more permanent, importance to its preservation or protection. Alternatively, to see darkness as instrumental makes it only valuable insofar as it achieves a pre-established end, and thus highly conditional. To say that darkness is valuable for sustainability or efficiency only remains true so long as darkness does in fact lead to energy reduction and cost savings. If new lighting technologies reach the same end of, say, a certain percentage reduction in energy usage, that goal is presumably satisfied regardless of any increase in darkness. The same could be said for achieving the health-related benefits of darker nights. Because of this, instrumental valuations of darkness are likely much less robust, and contingent upon technological developments.

**Fig. 1:**
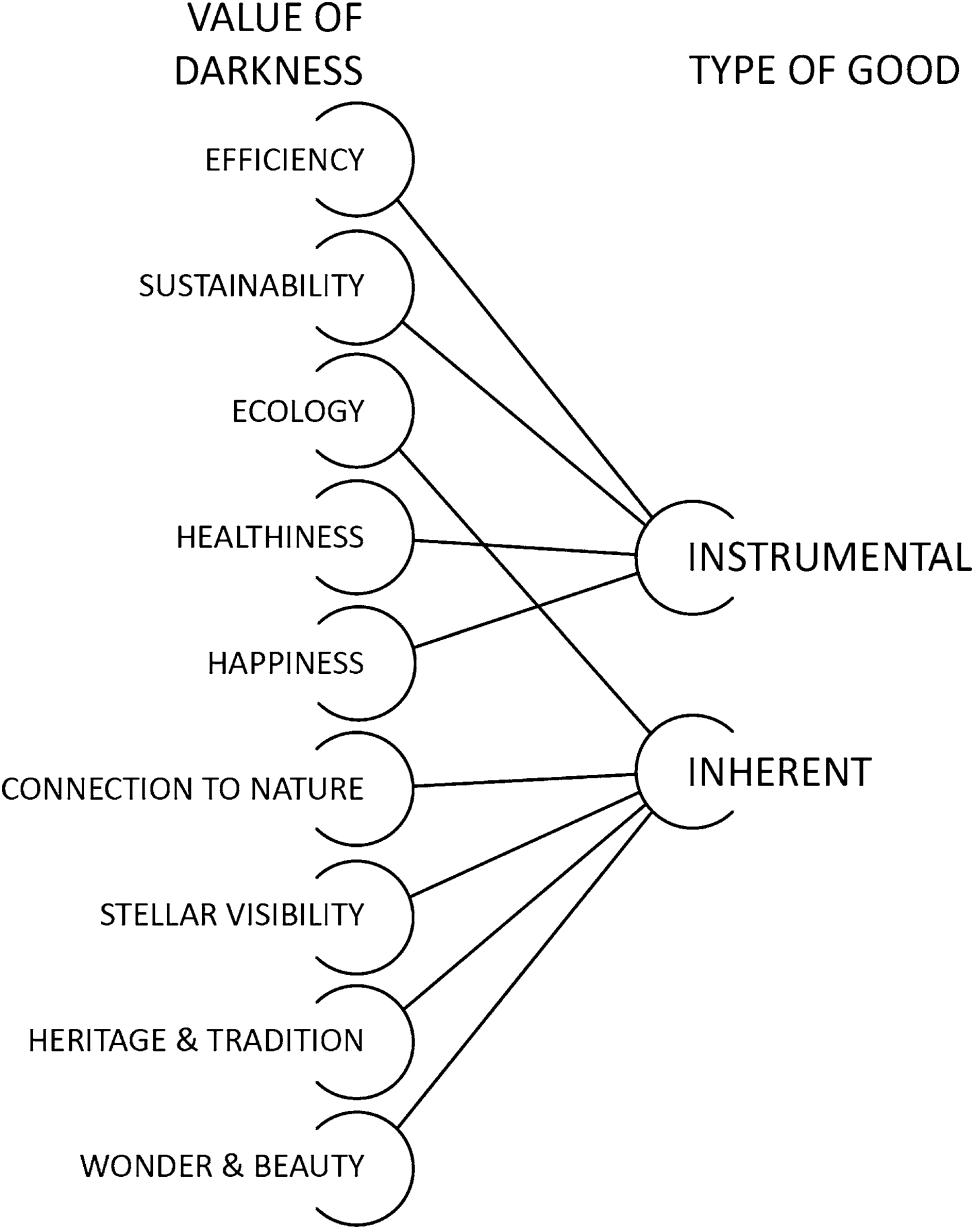
The value of darkness categorized by type of good

Thus, the valuations of darkness can be categorized into two broad types of goodness: *inherent* and *instrumental* (Fig. 1). However, while each valuation has been placed into a single category, this does not imply mutual exclusivity. Rather, these categories can be understood as qualitative lenses that clarify the meaning (and importance) of darkness in relation to desired ends.

### Temporal Characteristics

The temporal nature of environmental problems has become a salient topic in environmental ethics. But compared to other environmental concerns, the effects of light pollution do have somewhat unique temporal characteristics. The first is that, despite the label of “pollutant,” artificial light does not act in the same way. While the effects may linger, all the “pollution” can be effectively eliminated with a flick of a switch. Second is that the “loss of the night” does not have the same permanence as, say, the depletion of non-renewable resources or species extinction. The night—and by proxy darkness—is not actually lost, but rather access is hindered or obstructed. However, this does not necessarily make the effects of diminished darkness less important, or less impactful. We can consider the temporal characteristics of each valuation of darkness, and specifically how the longevity of their implied objectives relate.

For some valuations (e.g., efficiency, healthiness), arguments rest on the immediate, *present*-*oriented* benefits offered by a reduction in lighting and increase in darkness at night: it will save money today, it will improve our well-being now, etc. Other arguments have an ongoing outlook, seeking to mitigate certain effects over a period of several years or decades (e.g., energy reduction as a way to curb CO_2_ emissions, the protection of species and habitats). This, one could argue, is the temporal category of strongest moral concern, as these effects are largely irreversible. Thus, these valuations have been categorized as *imperatives* to signify that they have both an immediate and ongoing temporal importance. Still other arguments rest on a duty to protect access to the night sky for future generations, and ensure these meaningful experiences are recovered or preserved. For these arguments, there is often an assumption that many people have already lost, or are in the process of rapidly losing, these features of dark nights—an assumption supported by the world atlas of artificial night sky brightness mentioned in the introduction (Falchi et al. 2016). Actions taken now can therefore reverse or halt the disappearance of the night sky. As such, these have a *future*-*oriented* temporal characteristic.

**Fig. 2:**
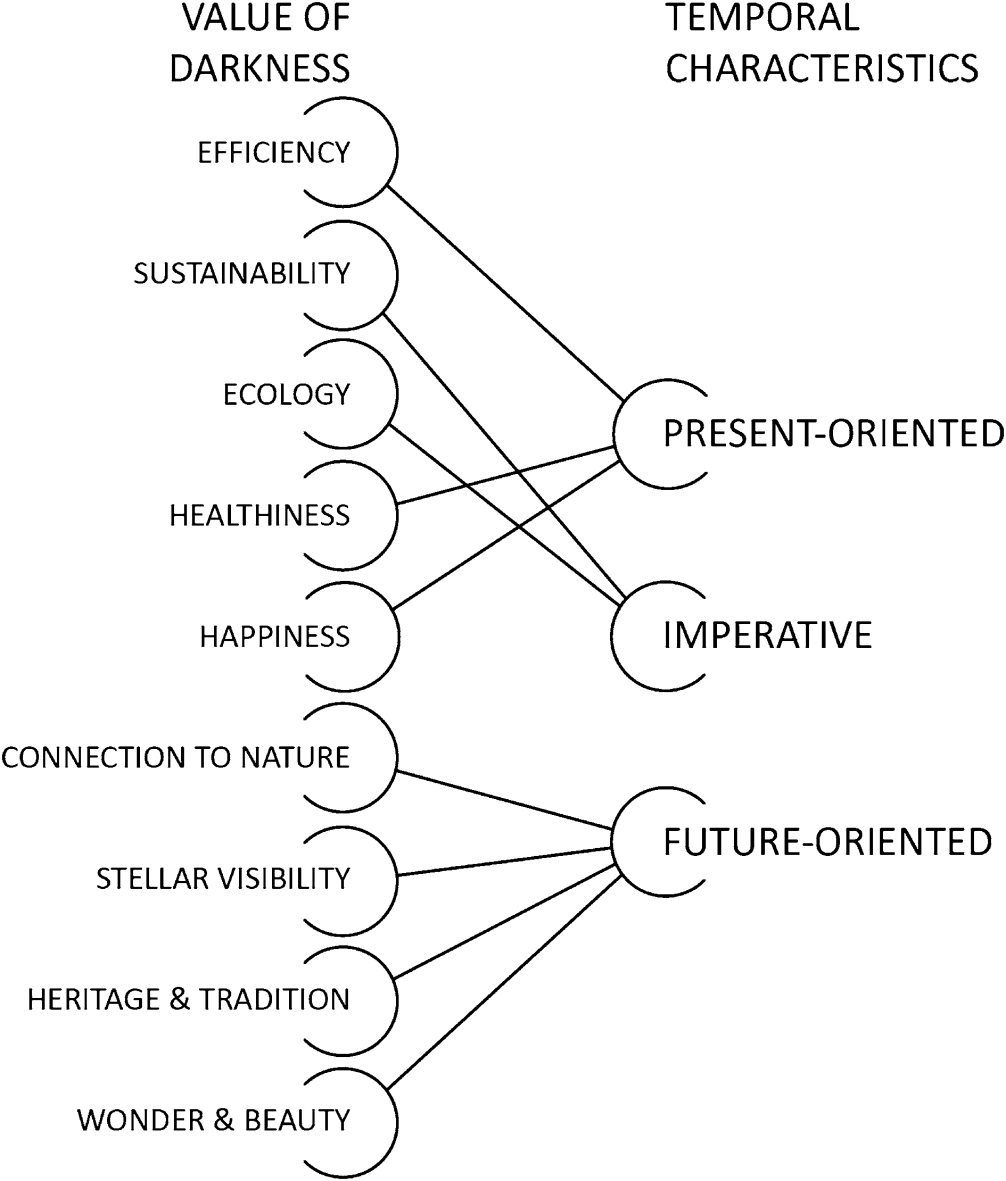
The value of darkness categorized by temporal characteristics

Thus, the valuations darkness can be further categorized into three broad temporal outlooks: *present*-*oriented*, *imperative,* and *future*-*oriented* (Fig. 2).

### Spatial Characteristics

In additional to temporal characteristics, spatial considerations are important for understanding the value of darkness. As discussed in the section “Articulating the value of darkness”, darkness here is considered as both an abstract concept and a tangible, accessible nighttime feature. Like other environmental considerations it can be directly experienced, and it is often via that direct experience that the act of valuing is derived. In this sense, exactly *where* the achievement of darkness is most valuable can be considered. For health-related impacts, as an example, it is most important that the places inhabited—our directly experienced surroundings—are dark. So, healthiness has a *terrestrial* characteristic. Not unsurprisingly, values related to the night sky take on a different spatial emphasis. For stellar visibility, as an example, the lighting quality of a streetscape is of little importance, so long as it is designed so that minimal skyglow is produced. Therefore, an *atmospheric* spatial characteristic for some valuations can also be identified. Interestingly, Gallaway (2014) identifies features associated with dark skies as most pertinent for fostering happiness, which gives this value an atmospheric spatial orientation. Ecological value derived from darkness is not easily categorized into one of these two spatial realms, as the identified effects of ecological light pollution (for example “disorientation”) have been attributed to both atmospheric skyglow and ground-level street lighting (Longcore and Rich 2004). At a general level, the ecological value of darkness encapsulates both spatial categories.

**Fig. 3:**
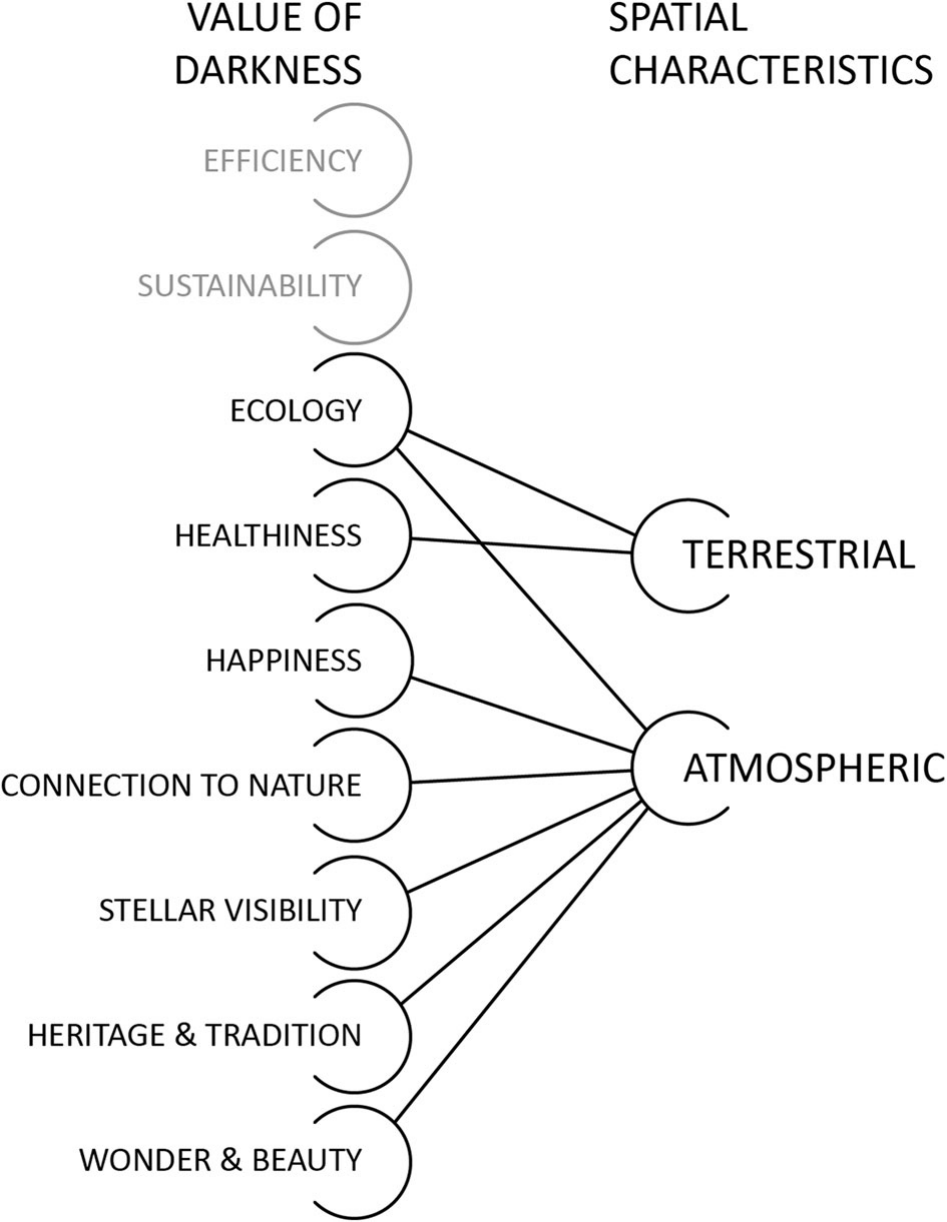
The value of darkness categorized by spatial characteristics

Thus, valuations of darkness can be further categorized into two broad spatial scales: *terrestrial* (*concerned with localized environmental conditions*), and *atmospheric* (*concerned with dark skies*) (Fig. 3). However, these categories should not be seen as mutually exclusive. The achievement of darker streets will often create darker skies (and vice versa). This distinction is meant to highlight where, spatially, the focal point of concern is situated. Interestingly, this categorization does not seem to hold for all instrumental valuations. Efficiency and sustainability, when understood via darkness, are most concerned with questions of quantity rather than spatial characteristics.

## Applying the Value of Darkness: from Evaluation Towards Prescription

The section “Categorizing the value of darkness” provides a systematic analysis of the environmental value of darkness. With this evaluation and categorization of darkness in hand, one can reflect on the implications for normative assessments of—and subsequent decision-making about—artificial nighttime lighting. Most would agree that each of the nine values discussed above is worthy of promotion or preservation, when considered abstractly. However, it is currently far less common to assign these (positive) values to darkness at night. In doing so, this analysis offers a way to engage with, and appreciate, the positive potential of darkness at night. It can help with asking just how much darkness is wanted in our urban nightscapes, and what exactly the preservation or reintroduction of darkness is trying to achieve. The following section presents the potential operationalization of this framework in broad terms (“Darkness as a set of prima facie obligations”) and via a brief case study (“Darkness as a prescriptive tool: LED streetlights”).

### Darkness as a Set of Prima Facie Obligations

Each of the nine values of darkness represents an important goal that darkness helps to preserve, protect, or promote. In considering how to incorporate these values into design and policy decisions, a *prima facie* moral obligation can be derived from each identified value (Table 2). To call these nine values a prima facie obligation is to say that there is a morally relevant reason for us to perform actions that uphold or strive for their achievement, and that “were it the only morally relevant feature of my situation, then the act in question would be my duty proper” (Timmons 2013, 249).^3^ As a prima facie obligation, the achievement of each value in every situation is not categorical. But, there is a duty to see each as an obligation that *should* be achieved if possible, and this comes with important implications. If it is accepted that darkness is worthy of moral consideration and is valuable in some ways, and that these nine obligations encapsulate the environmental value of darkness, and allow their translation into prima facie duties, then there is an important switch in the burden of proof. It becomes one of showing why it is good, or better, *not* to not promote, preserve, protect, or pursue some aspect of the value of darkness. There is a responsibility to incorporate these obligations, and the values they represent, into future decision-making about nighttime lighting.

**Table 2:** The environmental value of darkness articulated as prima facie obligations

Value of darkness	Prima facie obligation derived from value
Efficiency	The responsible use of lighting where and when needed; money-saving
Sustainability	The responsible use of lighting where and when needed; energy-saving and preserving non-renewable resources
Ecology	The protection and preservation of species and biodiversity; habitat conservation efforts
Healthiness	Promoting and fostering human health; physiological well-being
Happiness	Promoting and fostering happiness; emotional well-being
Connection to nature	Preserving a connection to the more-than-human world
Stellar visibility	Preserving conditions for access to the firmament
Heritage and tradition	Preserving the cultural heritage of the night sky for future generations
Wonder and beauty	Preserving the aesthetic appeal of the natural night sky

These nine obligations encapsulate the environmental value of darkness. Whether each can be achieved at once, or are in fact equally desirable in every instance, is a practical and procedural question. A way to conceive of the operationalization of these obligations is as a set of second-order moral obligations, where one *ought* to uphold them even if it is currently not possible to achieve all nine simultaneously (van den Hoven et al. 2012). There is then an increased responsibility for designers, engineers, and policy-makers to strive for innovations that make possible the full landscape of values, to avoid the problem of *moral overload*. Such problems occur when there are conflicting values or obligations that cannot all be satisfied at the same time. It has been argued that, in situations of moral overload, if we can bring about future change via innovation to satisfy all conflicting values or obligations, then there is a moral obligation to develop technologies towards this goal (van den Hoven 2013). Finding solutions that can accommodate the multi-faceted value of darkness, in combination with other values important to the use and enjoyment of urban nightscapes, becomes a primary design goal for nighttime illumination.

**Fig. 4:**
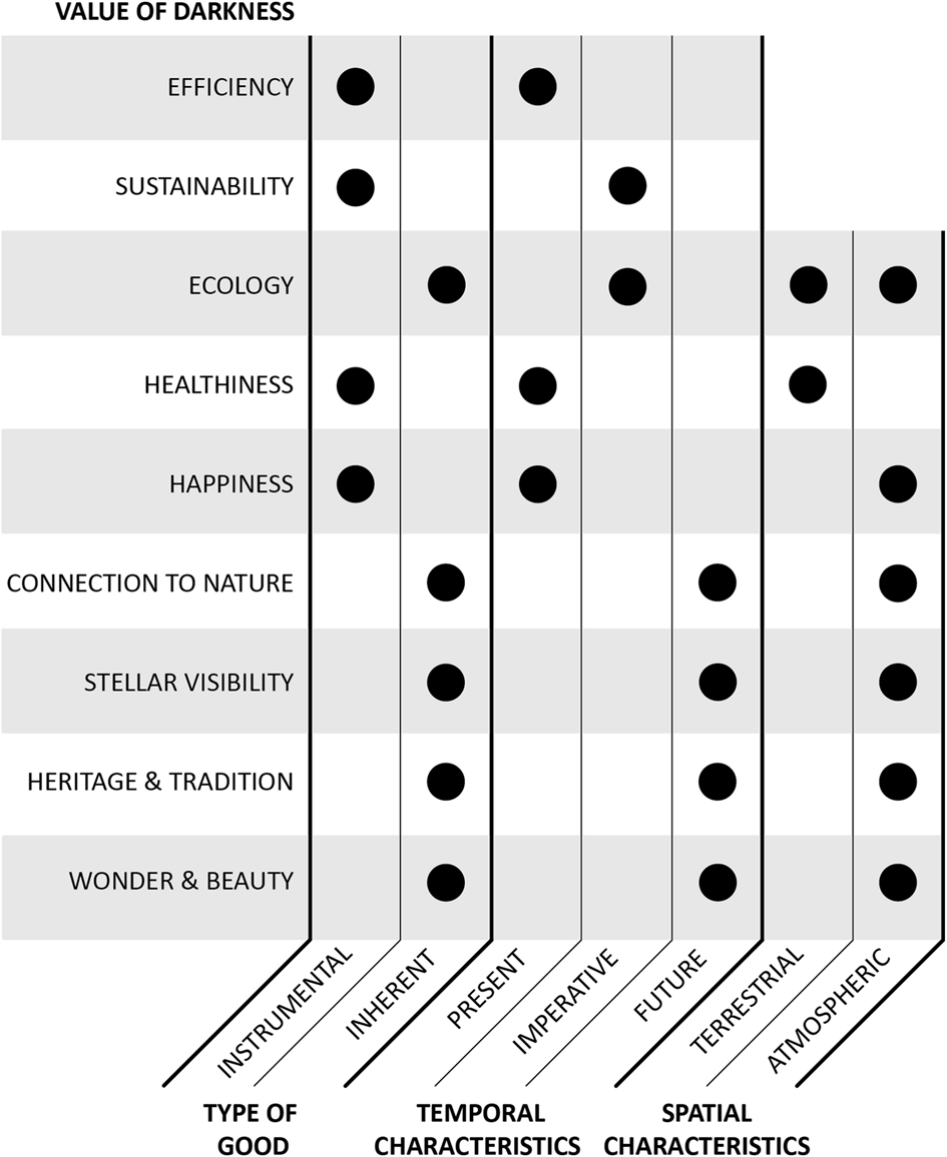
The value of darkness categorized by type of good, temporal characteristics, and spatial characteristics

How can steps be taken towards achieving such innovations? The analysis in the section “Categorizing the value of darkness” gives some direction for starting points by identifying interconnections and mutually reinforcing facets of darkness. Within this brief categorization, two general clusters can be seen emerging: present-oriented instrumental valuations of darkness, and future-oriented intrinsic valuations of dark skies (Fig. 4). The instrumental valuations are somewhat dispersed in their goals, however it seems—perhaps unsurprisingly—that the intrinsic valuations associated with the night sky (*connection to nature, stellar visibility, heritage and tradition,* and *wonder and beauty*) are much more closely intertwined. Within this cluster are a series of reciprocal relationships, as achieving any one of these goals creates conditions for the others to be met. While not adhering to as strict of a spatial boundary, *ecology* can also be seen as closely aligned with this cluster. Furthermore, inherent future-oriented values appear very likely to accommodate instrumental, present-oriented goals. Achieving something like a stronger *connection to nature* will likely lead to an increase in *efficiency* and *sustainability*, given the necessary reduction in brightness required for darker skies. However, it far less obvious if the opposite holds true. More work can be done to understand these interconnections, but it seems that from a conceptual viewpoint there is reason to focus on those values (and related obligations) that are inherent to darkness.

### Darkness as a Prescriptive Tool: LED Streetlights

The usefulness of the above framework can be shown via a brief and preliminary look at the growing adoption of LEDs for outdoor lighting. From the perspective of this framework, it would appear that the widespread retrofitting of street lamps with brighter, whiter LEDs is a shortsighted design and policy choice. While championed by many due to their energy-saving potential, longer lifetime, and improved visibility (e.g., De Almeida et al. 2014), the implementation of LEDs has been criticized for their potential environmental, health-related, and aesthetic consequences (e.g., IDA 2014). It appears that, while potentially saving money and energy in the short term [although even the reductions in cost and energy consumption from efficiency improvements to lighting has been debated due to the “rebound effect” (Kyba et al. 2014)], they may exacerbate other negative effects of light pollution. There is growing evidence that blue-rich LED lighting may increase skyglow (Morgan-Taylor 2014), thus decreasing access to dark skies. Furthermore, the health effects of LEDs are likely much worse for both humans and wildlife (AMA 2016). While colour adjustment may decrease the atmospheric effects, studies suggest this may not decrease the adverse ecological impacts (Pawson and Bader 2014).

It appears that the current usage of LEDs runs into the problem of moral overload, only satisfying a narrow interpretation of the value of darkness. While fulfilling the values of *efficiency*, and perhaps *sustainability*, they likely have negative effects on the other seven environmental values of darkness. Furthermore, a focus on dark skies alone may not address health or ecological concerns. In their present usage LEDs only satisfy an instrumental, present-oriented conception of darkness, and in doing so provide an incomplete solution to the problems of light pollution. Put otherwise, they satisfy two prima facie obligations at best. This does not imply a universal condemnation of LEDs, but does show that current strategies can be improved if a value-sensitive approach is adopted. The qualities of controllability and efficiency that make LEDs appealing can be utilized to foster and promote a wider range of desired goals (e.g., Gaston et al. 2012). By accounting for the value of darkness, emerging downstream issues can be avoided.

## Conclusion: Designing with Darkness

This paper provides a first comprehensive, systematic analysis of the value of darkness as a moral framework for evaluating urban nighttime lighting. Darkness has been conceptualized as an environmental good via a pluralistic definition informed by contemporary research into light pollution, and further categorized by type of good, temporal outlook and spatial characteristics. Prima facie obligations were derived from the value of darkness, which provide a value-sensitive starting point for new innovations and policy choices. Furthermore, a brief discussion of LED streetlights shows how this analysis can be further developed and applied as a prescriptive tool for decision-making about nighttime lighting.

The analysis of darkness presented here is a first step, but certainly should not be the last. In conceiving of darkness as something worth pursuing in our urban nightscapes, future developments must remain cognizant of its origins. The very foundations of valuing darkness from an environmental perspective—at least in its present form—is transitory. It is part reactionary, part proactive. And any achievement of “more darkness” will inevitably have consequences on its future conception, as well as broader understandings of cities at night. The challenge now for designers, innovators, and policy-makers is to incorporate darkness back into our nights without marginalizing other values inherent to, and inherited in, our nighttime lighting.

More work is needed to understand how darkness can be re-introduced into urban nightscapes in ways that do not denigrate or hinder values tied to lighting, such as safety and security, accessibility, nightlife, 24-hour societies, civic expression, etc. For example, the relationship between this framework and safety—in particular the complex dynamics of nighttime illumination, perceptions of safety, and actual safety—is an important topic for future research. Despite assumptions that brighter lights create safer nights, studies have reached contradictory conclusions regarding what level of lighting actually reduces traffic accidents and crime, and question whether lighting is the most pertinent factor to consider (Gaston et al. 2015; Henderson 2010). However, nighttime lighting has long been symbolically connected to safety and security (Schlör 1998), and a fear of the dark is arguably an innate human quality (Ekirch 2005). Further work towards understanding how this framework intersects with research on nighttime safety, and feelings of safety (e.g., Boomsma and Steg 2012; Haans and de Kort 2012), is paramount for determining design possibilities that are socially acceptable.

In addition to addressing potential value conflicts, other positive aspects of darkness at night can be considered alongside this framework. This includes non-environmental reasons for valuing darkness, such as intimacy, privacy, and anonymity. And for all these factors localized contexts should be further explored, as the geography, culture, and perspectives of local stakeholders will likely lead to different norms and design requirements. Operationalizing the value of darkness in localized settings will also bring to the fore important procedural considerations for including stakeholders in decision-making processes, as well as bring clarity to questions of just how much darkness is acceptable (and why). In sum, how the environmental value of darkness should be operationalized and put into dialogue with other values and needs related to nighttime lighting is a task for future research.

As a final thought, I would like to return to an idea that began this paper, namely re-framing the moral issue of light pollution. Utilizing the value of darkness to inform our decision-making offers a framework that encapsulates, but goes beyond, simply dealing with the negative effects of light pollution. It asks that we reconsider darkness, not as an opponent of lighting, but as an equal consideration in the design of nighttime spaces. And with this comes new opportunities, especially in cities. Edensor (2015, 436), in reflecting on the evolving perception of darkness in cities, states, “Rather than being lamented, the reemergence of urban darkness, although not akin to the medieval and early-modern gloom that pervaded city space, might be conceived as an enriching and a re-enchantment of the temporal and spatial experience of the city at night.” New possibilities lie ahead if we can design not just for less light pollution, but start designing with darkness.

## Footnotes

1 The details and figures on light pollution will not be discussed further in this paper, except as needed to support the different values of darkness presented in the “Categorizing the value of darkness” section. However, elsewhere I present a summary of the causes and effects of light pollution (Stone forthcoming). Much of this data can also be found on the website of the International Dark-Sky Association (darksky.org), as well as in articles by Henderson (2010) and Pottharst and Konecke (2013).

2 Two works have been particularly useful in establishing this list. The first is Gallaway’s “The Value of the Night Sky” (2014), which categorizes the instrumental value of the night sky in relation to the goals of happiness and sustainability. The second is Bogard’s *Let There Be Night* (2013), a detailed investigation into the intrinsic and instrumental value of natural nights.

3 While not explicitly referenced, it should be noted that this section invokes a Rossian approach (Ross 2002). While I do not endorse Ross’s specific list of prima facie duties or intrinsic goods, I do apply his notion of prima facie duties to translate the value of darkness into moral obligations.
